# Do the charges matter?—balancing the charges of the chromodomain proteins on the nucleosome

**DOI:** 10.1093/jb/mvz004

**Published:** 2019-01-15

**Authors:** Kyoko Hiragami-Hamada, Jun-ichi Nakayama

**Affiliations:** 1 Division of Chromatin Regulation, National Institute for Basic Biology, Okazaki, Aichi, Japan; 2 Department of Basic Biology, School of Life Science, SOKENDAI (The Graduate University for Advanced Studies), Okazaki, Aichi, Japan

**Keywords:** chromodomain, heterochromatin, histones, histone methylation, nucleosome

## Abstract

The chromodomain (CD) is a member of the Royal family of conserved chromatin-binding motifs with methylated substrate binding ability, and is often found in ‘readers’ or ‘writers’ of repressive histone marks. The regions upstream or downstream of the CD are generally highly charged. Several previous studies suggested that these charged regions modulate the CD’s chromatin-binding activity. Considering the relatively weak interaction between the CD and a modified histone tail, it is puzzling how the highly charged CD-flanking regions are ‘balanced’ on the highly charged nucleosomes to mediate a modification-dependent interaction. Interestingly, the charge distributions along the CD and surrounding regions appear to be distinct among different types of readers and writers, indicating their functional relevance. Here, we describe and discuss the current understanding of the highly charged CD-flanking regions and the potential experimental concerns caused by the regions.

Our genetic information is stored and regulated in the form of chromatin, a large, sophisticated multimeric complex composed of DNA, RNA and proteins. Chromatin folds itself into higher-order structures and provides the key regulatory mechanism for many chromatin-based biological processes, including transcription, DNA replication and DNA repair, by modulating the accessibility of factors to their target sites on DNA. The basic repeating structural and regulatory unit of chromatin is the nucleosome, which consists of 147 bp of DNA wrapped around a core histone octamer (two each of H2A, H2B, H3 and H4) (1). Each core histone possesses an N-terminal tail that extends from the nucleosome core. Covalent chemical modifications of the N-terminal tails act as a signal for recruiting effector proteins that alter or stabilize a particular chromatin structure (2). One of the intriguing facts about the nucleosome is how highly positively charged (*i.e*. basic) histone tails and highly negatively charged (*i.e*. acidic) DNA ‘balance’ themselves within or among nucleosome particles. Such opposite charges on histone tails and DNA have been implicated in both inter-nucleosome interactions and intra-nucleosomal histone tail-DNA interactions (3). Neutralization of positive charges or introduction of negative charges on histone H3/H4 tails, by acetylation or phosphorylation, respectively, weakens the histone tail–DNA interactions (3, 4). This leads to chromatin decompaction and/or an increase in the histone tails’ accessibility and modifiability (3, 4). Thus, the opposite charges on histone tails and DNA and their modulation have a considerable impact on chromatin structure.

To date, a plethora of histone modifications and so-called ‘reader’ proteins that recognize and bind to a specific histone modification(s) have been identified. In particular, di- and trimethyl histone H3 lysine9 (H3K9me2/3) and trimethyl H3K27 (H3K27me3) are ‘read’ by a member of the chromodomain (CD) protein family to form or maintain condensed chromatin structures, known as heterochromatin, for transcriptional repression and/or genome stability (5). Interestingly, most of the CD proteins and several members of the Tudor domain (TD) protein family, which also read H3K9me2/3 marks, have highly charged, flexible regions around the chromatin-binding CD/TD domain (Table I). Since the interaction between a modified histone tail and a CD is rather weak (μM range), the highly charged CD-flanking regions are thought to contribute to additional affinities for the CD protein–nucleosome interaction. As shown in Table I, the different types of CD/TD proteins have fairly distinct charge distributions along the CD/TD and flanking regions. The HP1-type methyl H3K9 reader proteins generally possess an acidic N-terminal region, an acidic-neutral globular CD, and a highly basic C-terminal hinge region. The acidic patch of the N-terminal region reportedly provides additional interaction surfaces for HP1 and the histone H3K9me3 tail (19, 20), whereas the basic hinge generally mediates nucleic acid binding (6, 21–23). Although the mechanism remains elusive, these oppositely charged N- and C-terminal regions of the CD seem to communicate and compensate for each other’s charge effects (19, 22). The polycomb (Pc)-type methyl H3K9/K27 reader proteins also follow a similar charge distribution pattern as the methyl H3K9 readers, but have a highly basic CD (24). This may account for the fact that most of the Pc-type CDs possess RNA/DNA-binding activity (17). The N-terminal regions of the Pc-type methyl H3K9/K27 readers are acidic, but their length is approximately half of that of the N-terminal regions of the HP1-type methyl H3K9 readers (10–11 versus 19–20 a.a.), and there is no stretch of acidic amino acids immediately adjacent to the CD. Therefore, the impact of the acidity of the N-terminal region is likely to be smaller in the Pc-type methyl H3K9/K27 readers, as compared with the methyl H3K9 readers. Interestingly, the histone-binding domains of methyl H3K9 writers also appear to be surrounded by highly charged regions. The CD of the SUV39 family and the TDs of SETDB1 are flanked by basic N- and C-terminal regions. The basic nature of these regions may contribute to the recruitment or stable binding of these enzymes to/with chromatin, by mediating the interaction with nucleic acids. Like the Pc-type CDs, the basic CDs of SUV39H1/Suv39h1 and the yeast homologue Clr4 show RNA-/DNA-binding activity, whereas Suv39h2, with an acidic CD, seems to interact with nucleic acids through its basic N-terminal region (14, 25–27). Taken together, the charge distributions along the CD/TD and their surrounding regions may serve as a good predicting factor for the binding specificity and functional properties of the CD/TD proteins.

**Table I.** **Summary of the pI values of the chromatin-binding domains and their flanking regions of human and fission yeast methyl H3K9/K27 readers** ^a^

Type	Protein and chromatin-binding domain	N-terminal	CD/TD	Downstream/Hinge^b^	Specificity
HP1	CBX5 CD	4.66	6.8	10.21	methyl H3K9 (6)
HP1	CBX1 CD	4.69	4.67	7.98	methyl H3K9 (6)
HP1	CBX3 CD	6.1	4.68	10.11	methyl H3K9 (6)
HP1	Swi6 CD (*S. pombe*)	4.7	5.59	9.52^b^	methyl H3K9 (7, 8)
HP1-like	Chp1 CD (*S. pombe*)	3.66	6.42	11.2^b^	methyl H3K9 (9)
HP1	Chp2 CD (*S. pombe*)	4.51	4.05	9.88	methyl H3K9 (8)
MPP8	MPP8 CD	3.5	5.37	9.45	methyl H3K9/K23 (10, 11)
UHRF1	UHRF1 TD	4.04	5.16	8.58	methyl H3K9 (12)
H3K9MTase	SUV39H1 CD	8.73	8.64	10.09	methyl H3K9 (13)
H3K9MTase	SUV39H2 CD	8.88	5.66	9.56	methyl H3K9 (14)
H3K9MTase	Clr4 CD (*S. pombe*)	4.53	9.80	10.62	methyl H3K9 (15)
H3K9MTase	SETDB1 Triple TDs	9.46	7.82	10.55	methyl H3K9-acetyl K14 (16)
Pc	CBX2 CD	3.67	9.52	11.9	methyl H3K9/27 (17)
Pc	CBX4 CD	4.51	9.52	11.84	methyl H3K9/27 (17)
Pc	CBX6 CD	4.53	9.45	11.17	Methyl H3K27? (17)
Pc	CBX7 CD	3.79	9.3	9.66	methyl H3K9/27 (17)
Pc	CBX8 CD	4.53	8.27	10.13	methyl H3K27 (17)
CDYL	CDYL1a CD	9.99	6.33	11.59	? (18)
CDYL	CDYL1b CD	3.67	6.33	11.59	methyl H3K9/K27 (11, 18)

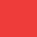
pH < 4 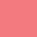
pH 4 – 6 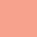
pH 6 – 7 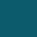
pH 7 – 8 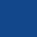
pH 8 – 9 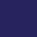
pH > 9

^a^The acidity and alkalinity of each region are shown in different shades of red and blue, respectively. For some CD/TD proteins with long N- or C-terminal regions, the first 40–50 amino acids immediately adjacent to the CD were subjected to the pI analysis.

^b^Since the hinge region of HP1-type proteins is long and charge-biased within the region, the pI values of the first 50 amino acids immediately downstream from the CD are shown here. The pI values of the full hinge and downstream region of Swi6 and Chp1 are 5.56 and 5.40, respectively.

Previous biochemical or biophysical studies often employed synthetic peptides or DNA/RNA to investigate the interactions between the CD proteins and ‘chromatin’. These studies have provided significant insights into the binding specificity and/or the DNA/RNA binding activity of the CD proteins. However, can the use of one component of the nucleosome, which is likely to over-represent either acidic or basic charges, truly reflect the binding of a CD protein to chromatin? Or can the observations made with the individual components of the nucleosome be summed up into the context of the whole nucleosome? The same questions apply to a CD protein if a truncated version of the protein is used in an assay: the addition or omission of the upstream or downstream regions of the CD may introduce unnatural charge biases. In other words, an inappropriate combination of charge-biased ‘prey and bait’ may result in a misleading observation. In order to avoid complications, the use of designer nucleosomes, containing a histone/DNA modification of interest and a full-length CD protein or a CD with both N- and C-terminal flanking regions, is probably the most ideal *in vitro* model system to study the biochemical and biophysical properties of the CD protein - chromatin interaction. However, it must be noted that, as Kujirai *et al.* (28) have demonstrated, contamination of the *in vitro* reconstituted nucleosomes with free histones, as well as the saturation level of oligonucleosomes, significantly influences the biophysical behaviours of the nucleosomes. Furthermore, the presence of free histones or under-saturated oligonucleosomes with a high nucleosome-free DNA content may interfere with the modification-dependent association of a CD protein with nucleosomes, by prematurely or non-specifically interacting with the charged regions of the CD proteins (19, 29). Consequently, appropriate purification and quality monitoring of the reconstituted nucleosomes are quite essential.

Although the pI values can provide a rough idea of how the adjacent regions affect the CD’s activity, there are several cases that do not fit the general rules. For example, the CBX3 hinge is as basic as the CBX5 hinge, but only the latter exhibits strong nucleic acid binding activity (21–23). Another example is the CBX2 CD, which is highly basic like other Pc-type CDs, but does not seem to bind RNA/DNA (17). Thus, in some cases, the pI value alone cannot predict the charge-based behaviour of the protein region, and additional factors, such as the length or 3D presentation of the charged amino acids, may determine the effects. Last but not least, a subset of the CD proteins is subjected to posttranslational modifications, including charge-modulating phosphorylation and acetylation *in vivo* (30, 31). Phosphorylation of the CD-flanking regions has been described to have negative effects on the nucleic acid binding of several CD proteins (22, 31). Nevertheless, further investigations of other CD proteins are required to generalize the effects exerted by charge-modulating modifications.

In summary, both nucleosomes and CD proteins comprise highly charged molecules or regions. It is unclear if the binding of the CD proteins affects the local charge compensatory mechanisms of the nucleosomes. Depending on the location and number of modified sites, charge-altering posttranslational modifications of histones and CD proteins can potentially exert significant effects on their interactions, and thus on the chromatin structure. Future biochemical and biophysical studies on various CD proteins with charge-altering mutations or modifications are expected to provide deeper mechanistic and functional insights into the roles of the charged CD-flanking regions.

## Funding

This research was funded by MEXT/JSPS KAKENHI grant numbers JP17K07293 (to K.H-H.), JP23114005 (to J.N.), JP16H01315 (to J.N.) and JP18H05532 (to J.N.).

## Conflict of Interest

None declared.

## Abbreviations

CD: chromodomain
Pc: polycomb
TD: Tudor domain
